# Sodium Glucose Co-Transporter 2 Inhibition Does Not Favorably Modify the Physiological Responses to Dietary Counselling in Diabetes-Free, Sedentary Overweight and Obese Adult Humans

**DOI:** 10.3390/nu12020510

**Published:** 2020-02-18

**Authors:** Shane P.P. Ryan, Alissa A. Newman, Jessie R. Wilburn, Lauren D. Rhoades, S. Raj J. Trikha, Ellen C. Godwin, Hayden M. Schoenberg, Micah L. Battson, Taylor R. Ewell, Gary J. Luckasen, Laurie M. Biela, Christopher L. Melby, Christopher Bell

**Affiliations:** 1 Department of Health and Exercise Science, Colorado State University, Fort Collins, CO 80523, USAAlissa.Newman@colostate.edu (A.A.N.); jes0513@gmail.com (J.R.W.); Raj.Trikha@rams.colostate.edu (S.R.J.T.); Hayden.Schoenberg@colostate.edu (H.M.S.); Taylor.Ewell@rams.colostate.edu (T.R.E.); Laurie.Biela@colostate.edu (L.M.B.); 2 Department of Food Science and Human Nutrition, Colorado State University, Fort Collins, CO 80523, USA; lrhoades@foodbanklarimer.org (L.D.R.); ellengodwin19@gmail.com (E.C.G.); mbattson@msudenver.edu (M.L.B.); Chris.Melby@colostate.edu (C.L.M.); 3 Medical Center of the Rockies Foundation, University of Colorado Health, Loveland, CO 80538, USA; Gary.Luckasen@uchealth.org

**Keywords:** weight loss, diabetes, body composition, SGLT2

## Abstract

Sedentary obesity is associated with increased risk of many cardio-metabolic diseases, including type 2 diabetes. Weight loss is therefore a desirable goal for sedentary adults with obesity. Weight loss is also a well-documented side effect of sodium glucose co-transporter 2 (SGLT2) inhibition, a pharmaceutical strategy for diabetes treatment. We hypothesized that, compared with placebo, SGLT2 inhibition as an adjunct to out-patient dietary counselling for weight loss would lead to more favorable modification of body mass and composition, and greater improvement in glucose regulation and lipid profile. Using a randomized, double-blind, repeated measures parallel design, 50 sedentary men and women (body mass index: 33.4 ± 4.7 kg/m^2^; mean ± SD) were assigned to 12 weeks of dietary counselling, supplemented with daily ingestion of either a placebo or SGLT2 inhibitor (dapagliflozin: up to 10 mg/day). Dietary counselling favorably modified body mass, body fat, glucose regulation, and fasting concentrations of triglyceride and very low-density lipoprotein cholesterol (main effects of counselling: *p* < 0.05); SGLT2 inhibition did not influence any of these adaptations (counselling × medication interactions: *p* > 0.05). However, SGLT2 inhibition when combined with dietary counselling led to greater loss of fat-free mass (counselling × medication interaction: *p* = 0.047) and attenuated the rise in high-density lipoprotein cholesterol (counselling × medication interaction: *p* = 0.028). In light of these data and the health implications of decreased fat-free mass, we recommend careful consideration before implementing SGLT2 inhibition as an adjunct to dietary counselling for weight loss in sedentary adults with obesity.

## 1. Introduction

Two thirds of adults within the United States of America are overweight; approximately half of those who are overweight satisfy the criteria for obesity [1,2]. Recent predictive models suggest that the prevalence of obesity within the USA will continue to increase, with no reversal likely in the near future [3]. The clinical implications of this increase are not trivial. Obesity, and especially sedentary obesity, has been linked with greater risk of cancer, dementia, organ damage, and many cardio-metabolic diseases, including type 2 diabetes [4,5,6].

Perhaps as a result of the increasing prevalence of type 2 diabetes, during the previous decade there has been a surge in the development of new pharmacological therapies. Among these new therapies is a class of medications that work via sodium glucose co-transporter 2 (SGLT2) inhibition. Therapeutic benefits of SGLT2 inhibition include short-term and sustained favorable modification of glycosylated hemoglobin (HBA1c), and fasting blood glucose [7,8,9,10,11,12]. SGLT2 inhibition lowers circulating glucose concentration by attenuating renal glucose reabsorption and promoting glucosuria. The magnitude of glucosuria is approximately 40–80 g/day [13,14]. Noteworthy, SGLT2 inhibition-mediated glucosuria has been well documented in clinical populations [7,8] and also in adults who are free from diabetes [13,14]. One unintended but potentially favorable aspect of glucosuria and the reduction in absorbed dietary carbohydrates is negative energy balance, and short-term and sustained weight and fat loss; effects that have been the focus of several recent and informative reviews [15,16,17,18].

Weight loss is a desirable goal for sedentary adults with obesity for many health-related reasons, including decreasing the risk of developing type 2 diabetes [19,20]. Dietary counselling combined with moderate caloric restriction is a reasonably effective means by which to evoke short-term (20–48 weeks) weight loss [21,22]. In light of the weight loss associated with use of SGLT2 inhibitors, the purpose of the current study was to determine the efficacy of supplementing dietary counselling for weight loss with the SGLT2 inhibitor, dapagliflozin. That is, we sought to explore an off-label application of SGLT2 inhibition. We hypothesized that, compared with placebo, SGLT2 inhibition as an adjunct to dietary counselling would lead to greater magnitude of weight loss, more favorable modification of body composition, improved glucose regulation, and improved lipid profile. The study has significance for public health as it has the potential to augment weight loss strategies in sedentary adults with obesity and decrease the risk of developing diabetes.

Considering the influence of SGLT2 inhibition on the absorption of dietary carbohydrates, a secondary aim was to explore the effects of dapagliflozin and dietary counselling on dietary preferences and hunger and appetite prior to and following acute feeding. In recent studies, the influence of short-term (2 weeks) SGLT2 inhibition on dietary preferences (i.e., increased desire for sweet foods) and appetite did not attain statistical significance [23]. In the current study, we extended the period of SGLT2 inhibition to 12 weeks and hypothesized that this longer duration of treatment would evoke measurable effects on dietary preferences, and hunger and appetite.

## 2. Materials and Methods

### 2.1. Study Design

A double-blind, repeated measures parallel design was utilized. Stratified random assignment was used to allocate participants to treatment. The trial was conducted in accordance with the guidelines for Good Clinical Practice and was registered at ClinicalTrials.gov (Identifier: NCT03180489). All procedures were reviewed and approved by the Institutional Review Board at Colorado State University in accordance with the principles set out in the Declaration of Helsinki. All participants provided written informed consent before commencing involvement in the study.

### 2.2. Participants

Sedentary, but otherwise healthy, men and women volunteered and participated. Inclusion criteria included: provision of informed consent, aged between 18–65 years, body mass index (BMI) greater than 27.5 kg/m^2^, sedentary, as defined as less than or equal to three low-intensity physical activity sessions per week, of less than or equal to 30 min per session over the previous year, negative pregnancy test for women of childbearing potential, no known metabolic diseases, and willingness to modestly reduce daily caloric intake for twelve weeks. Exclusion criteria consisted of evidence of any significant disease that may interfere with study objectives, habitual use of herbal preparations or prescription drugs other than birth control and/or medications to treat depression, current enrollment in another clinical study, habitual or recent use of tobacco in the last two years, history of hypersensitivity reaction to SGLT2 inhibition, renal impairment (based on estimated glomerular filtration rate), abnormal liver function, bilirubin >2.0 mg/dL (34.2 μmol/L), diagnosis of hepatitis B or C, history of bladder cancer, recent cardiovascular events, or considered unsuitable for participation by our medical monitor.

### 2.3. Protocol Overview

Following screening, all research participants began 12 weeks of dietary counselling for weight loss, combined with daily ingestion of either a placebo or a SGLT2 inhibitor. Prior to and following 12 weeks of dietary counselling, participants were assessed for body mass and composition, resting metabolic rate, blood glucose regulation, plasma lipid profile, and hunger and appetite.

### 2.4. Procedures

Screening included review of medical history, physical examination, venous blood sampling, and analysis for standard markers of kidney and liver function (via automated enzymatic reactions and spectrophotometry, Piccolo Express, Abbott, Princeton, NJ, USA), and completion of an exercise stress test (from rest through to volitional fatigue) with 12-lead electrocardiogram and blood pressure assessment. Following participant enrollment, as detailed below, primary outcome variables were measured over several separate visits to the laboratory before and after dietary counselling.

Body composition was assessed using dual energy X-ray absorptiometry (DEXA: Hologic, Discovery W, QDR Series, Bedford, MA, USA), as previously described [24]. In addition, body mass was determined using a physician’s digital scale, and waist circumference was measured midway between the lower border of the costal margin and the uppermost border of the iliac crest. Peak oxygen consumption (VO_2peak_) was determined via indirect calorimetry (Parvo Medics, Sandy, UT, USA) during incremental stationary cycle ergometer exercise to volitional fatigue, as previously described [24,25].

Following a 12-h fast, and 24-h abstention from exercise and caffeine, participants reported to the laboratory for measurement of resting metabolic rate (RMR) and respiratory exchange ratio (RER), and completion of an oral glucose tolerance test (OGTT). RMR was measured over 45 min, as previously described [26,27]. The first 15 min were considered a habituation/relaxation period, thus the RMR measurement corresponded to data collected during minutes 15–45. VO_2_ and carbon dioxide production (VCO_2_) were averaged each minute for 30 min using a custom built ventilated hood indirect calorimetry system (Nighthawk Design, Boulder, CO, USA) that utilized a respiratory mass spectrometer (Perkin Elmer MGA 1100, MA Tech Services, St. Louis, MS, USA) and an ultrasonic flow sensor (ndd Medizintechnik AG, Zürich, Switzerland). The system was calibrated daily with precision mixed gases (Airgas, Denver, CO, USA). Energy expenditure was calculated using the Weir formula [28]. In our laboratory, the measurement of RMR has a coefficient of variation of 3.3% and a test re-test r^2^ of 0.93 [29].

Immediately following the measurement of RMR, participants ingested 75 g of glucose diluted in 300 mL water; venous blood was sampled prior to ingestion and systematically over the next two hours for the determination of circulating concentrations of glucose (2300 STAT Plus Glucose Lactate Analyzer, YSI Inc., Yellow Springs, OH, USA) and insulin (enzyme-linked immunosorbent assay (ELISA): Crystal Chem, Inc., Elk Grove Village, IL, USA) in a manner similar to that previously described [24,30]. Homeostasis model assessment (HOMA-IR) was calculated from fasting glucose and insulin values, as previously described [31]. In addition, venous blood was also sampled for determination of fasting lipid profile (automated enzymatic reactions and spectrophotometry, Piccolo Express, Abbott, Princeton, NJ, USA), and concentrations of glucagon prior to and following glucose ingestion (ELISA: Mercodia AB, Upsalla, Sweeden). Areas under the glucose, insulin, and glucagon response curves were calculated using the trapezoid rule.

On a separate day, participants underwent testing for assessment of hunger and fullness. Following a 12-h fast and 24-h abstention from exercise and caffeine, participants ingested a small mixed macronutrient liquid meal (i.e., a preload: 1 kilocalorie/kg body mass; Ensure: 24% fat, 60% carbohydrate, 16% protein; Ross Laboratories, Abbot Park, IL, USA). Sixty minutes after the liquid preload, participants selected food from an all-you-can-eat breakfast buffet. Participants were provided 20 min to eat. The selected food choices (packaging included) were recorded and weighed before and after participant consumption. Remaining uneaten food was reweighed and participants’ energy and macronutrient intakes were determined using the Food Intake Analysis System software (U Texas Health Sciences Center, Houston, TX, USA). Participants remained at the facility for the next three hours for follow up satiety measurements.

At baseline, 0 and 60 min after liquid pre-load consumption, and 0, 60, 120, and 180 min after breakfast, participants completed a hunger and satiety survey. Surveys consisted of five questions: (1) How strong is your desire to eat right now? (2) How hungry do you feel right now? (3) How full do you feel right now? (4) How much do you think you could eat right now? (5) How thirsty do you feel right now? Participants answered these questions by marking a vertical line on a 100 mm visual analog scale, as previously described [32].

### 2.5. Dietary Counselling

Participants completed a three-day diet log (two weekdays and one weekend day) before initiation of the 12-week nutrition education and counselling intervention. Diet logs included all food and drink consumed (ingredients and amounts), location of meal, time of day, and hunger level before meal. Commercially available software (NutritionistPro, Axxya Systems, Redmond, WA, USA) was used to determine the caloric and micro- and macronutrient make up of each item consumed. Self-reported dietary analyses are often inaccurate in quantifying energy and macronutrient intake [33,34], thus the 3-day food records were used primarily as information regarding participants’ food intake patterns as a starting point for nutrition education/counseling.

A 12-week outpatient dietary intervention program (a modified version of Healthy You^®^ developed by the Kendall Reagan Nutrition Center at Colorado State University) was initiated, which consisted of weekly, one-on-one, 30-min meetings providing nutrition education/counselling for the study participants. Counselling focused on modest caloric restriction with an initial target of RMR multiplied by an activity factor of 1.2 and then, as needed, lowered over time toward an energy intake closer to the measured baseline RMR. This dietary reduction was designed to reduce each participant’s weight by approximately 0.5 kg per week [32]. Modest rather than severe energy restriction was targeted so as to not overwhelm or mask the potential contribution of SGLT2 inhibition to the weight loss. The percentage of kilocalories from each of the three macronutrients was not prescribed but individuals were encouraged to maintain a healthy balance of complex carbohydrates from whole grains, fruits, vegetables, and legumes, ingest adequate protein to meet the recommended dietary allowance of 0.8 g/kg body weight, and to reduce animal fats. This approach was for the purpose of maintaining adequate micronutrient intakes in the face of the calorie reduction. Participants were taught methods to reduce their overall daily caloric intake to assist with weight loss while avoiding making extreme changes in diet nor adopting extreme dietary patterns such as the ketogenic or vegan diet. These suggestions included mindful eating (eating more slowly and identifying cues and stimuli associated with overeating), increasing weekly frequency of home-cooked meals while limiting commercially prepared foods and beverages high in added sugars and fats, provision of recipe suggestions, increasing daily fiber intake, and increasing daily water consumption. Additionally, participants were instructed to maintain current activity levels and not allowed to begin a program of exercise. During weeks 5 and 10, participants completed a surprise 24-h dietary recall for the purpose of identifying possible barriers to the prescribed dietary changes and to help create strategies to enhance dietary objectives.

### 2.6. Adjunct Treatment

Research participants were assigned to placebo or the SGLT2 inhibitor, dapagliflozin, using a stratified random process to balance the distribution of males and females between groups. Assignments were completed in a double-blind fashion. Oral ingestion of either dapagliflozin or placebo coincided with the first day of dietary counselling and ended on the last day of data collection. The dose of dapagliflozin began as 5 mg/day for the first 14 days. In the absence of complications, side effects, or unfavorable reactions, the dose was increased to 10 mg/day for the remainder of the study.

### 2.7. Statistical Analysis

Baseline (pre-counselling) differences between placebo and SGLT2 inhibition groups were compared using independent Student t-tests. Consistent with the experimental design (randomized, double-blind, parallel, repeated measures), the influence of SGLT2 inhibition on the physiological responses to dietary counselling was examined using two-way (group: placebo vs. SGLT2 inhibition × time: before vs. after dietary counselling) analysis of variance (ANOVA). The influence of SGLT2 inhibition and dietary counselling on the dynamic responses to oral glucose ingestion were examined using three-way ANOVA (placebo vs. SGLT2 inhibition × before vs. after dietary counselling × time points during the OGTT). Pairwise multiple comparison procedures were performed using the Holm–Sidak method. Relations of interest were described using Pearson product-moment correlations. The level of statistical significance was set at *p* < 0.05. Data are reported as mean and standard deviation, unless otherwise indicated. Calculations were performed using commercially available statistical software (SigmaStat 3.0, Systat Software Inc., San Jose, CA, USA).

## 3. Results

**Figure 1:**
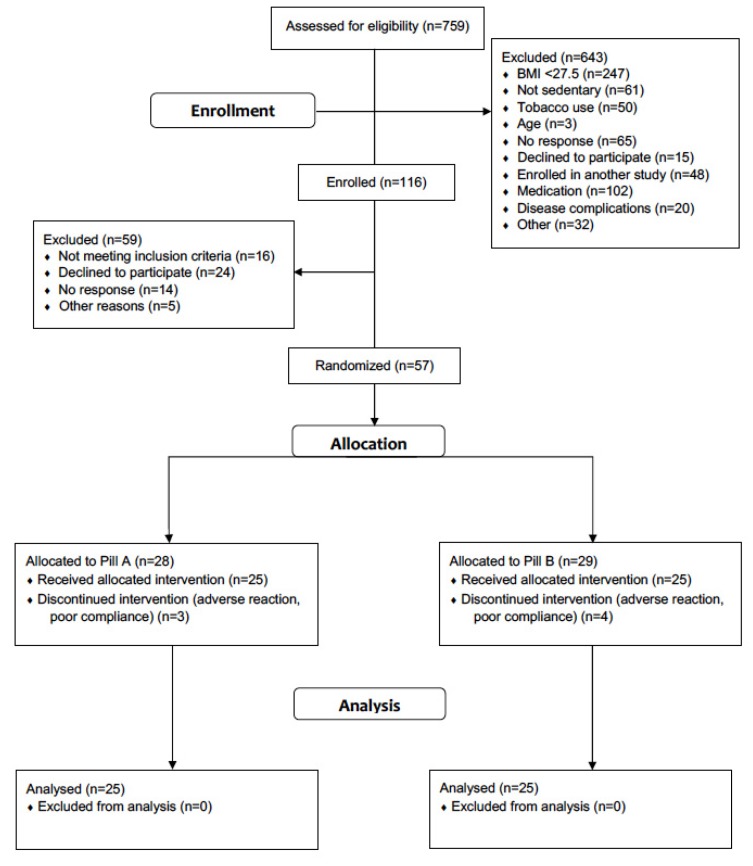
Progress of all participants throughout the trial (from screening through to completion).

The progress of all participants throughout the trial (from screening to completion) is presented in Figure 1. A total of 50 participants completed the study; baseline physiological characteristics are presented in Table 1. Consistent with the inclusion and exclusion criteria, participants displayed physiological characteristics typical of mostly sedentary adults with overweight or obesity. Body mass index at baseline was greater than or equal to 30 kg/m^2^ in 36/50 participants; the remainder of the participants ranged between 27.5 and 29.9 kg/m^2^. There were no baseline differences in any of the primary variables between the placebo and SGLT2 inhibitor groups (all *p* > 0.05). The dose of dapagliflozin began as 5 mg/day for the first 14 days and was increased to 10 mg/day for all participants for the remainder of the study. Baseline daily dietary intake data, calculated from three-day diet diary records, are also presented in Table 1.

Average daily dietary intake was greater in the placebo group (*p* = 0.015); this difference appeared to be mediated primarily by a greater carbohydrate ingestion (*p* = 0.012). Average daily ingestion of fat and protein was not different between placebo and SGLT2 inhibition (both *p* > 0.09).

### 3.1. Dietary Counselling

Overall attendance at dietary counselling sessions was not influenced by SGLT2 inhibition. That is, participants assigned to the dietary counselling supplemented with placebo attended the same number of counselling sessions as participants assigned to adjunct SGLT2 inhibition. Daily dietary intake, calculated from dietary recall, after five and ten weeks of dietary counselling are presented in Table 2. There were no main effects of treatment (placebo vs. SGLT2 inhibition), time (week 5 vs. week 10), or interactions (treatment × time) for any of the parameters, suggesting that the dietary counselling was consistent between groups.

### 3.2. Body Composition

Body mass and composition data are presented in Table 3. Following 12 weeks of dietary counselling, body mass was decreased in 45/50 participants. The change in body mass ranged between −16.0 and +2.3 kg. Overall, there was no difference in the magnitude of weight loss between participants assigned to placebo (−3.4 ± 4.1 kg) vs. SGLT2 inhibition (−4.3 ± 3.2 kg). With respect to body composition, fat mass, % body fat, and fat-free mass were decreased; SGLT2 inhibition did not influence any of these modifications with the exception of fat-free mass. The magnitude of decrease in fat-free mass was greater when dietary counselling was combined with SGLT2 inhibition (Placebo: −0.67 ± 2.51 vs. SGLT2 Inhibition: −1.98 ± 1.99 kg).

### 3.3. Resting Metabolism

Following 12 weeks of dietary counselling, RMR was decreased (main effect of time: *p* = 0.049); SGLT2 inhibition did not influence this response (Placebo 1636 ± 282 vs. 1543 ± 262 kcal/day; SGLT2 inhibition 1610 ± 235 vs. 1572 ± 310 kcal/day; group-by-time interaction: *p* = 0.397). Similarly, RER was also decreased (main effect *p* = 0.002) and the response unaffected by SGLT2 inhibition (Placebo 0.90 ± 0.05 vs. 0.86 ± 0.07; SGLT2 inhibition 0.88 ± 0.05 vs. 0.84 ± 0.06; *p* = 0.898). There were no group differences for either of these dependent variables (main effect of group: both *p* > 0.228).

### 3.4. Glucose Regulation

**Figure 2:**
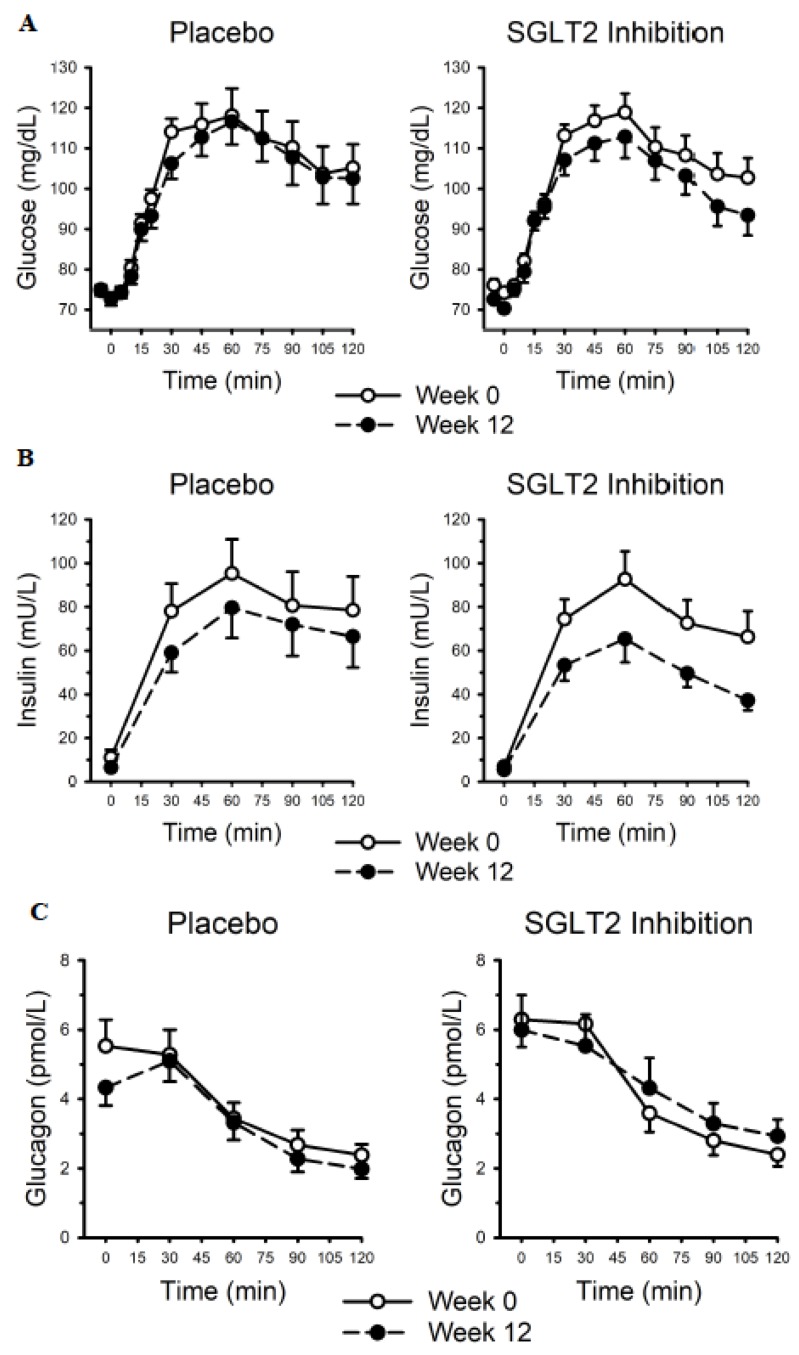
Circulating glucose (**A**), insulin (**B**), and glucagon (**C**) concentration during oral glucose tolerance testing prior to and following 12 weeks of dietary counselling for weight loss supplemented with placebo or sodium glucose co-transporter 2 (SGLT2) inhibition. Compared with week 0, glucose was lower during the week 12 oral glucose tolerance test (OGTT; main effect *p* = 0.004), but there was no group difference (main effect *p* = 0.458) or influence of SGLT2 inhibition (interaction *p* = 0.406). Overall, insulin was greater in the placebo group (main effect *p* = 0.032), and compared with week 0, insulin was lower during the week 12 OGTT (main effect *p* < 0.001); there was no influence of SGLT2 inhibition (interaction *p* = 0.382). On average, glucagon was greater in the SGLT2 inhibition group (main effect *p* = 0.023) but was unchanged over 12 weeks (main effect *p* < 0.582); SGLT2 inhibition did not influence this response (interaction *p* = 0.276). Data are mean and SE.

Fasting glucose was not different between groups (*p* = 0.751), was unchanged (*p* = 0.174) over 12 weeks, and SGLT2 inhibition did not influence the overall response (Placebo: 74.7 ± 6.9 vs. 75.0 ± 7.8; SGLT2 Inhibition: 76.1 ± 7.7 vs. 72.6 ± 5.5 mg/dL; *p* = 0.108). Circulating glucose concentration during the OGTT is presented in Figure 2A. Compared with week 0, glucose was lower during the week 12 OGTT (main effect *p* = 0.004), but there was no group difference (main effect *p* = 0.458) or influence of SGLT2 inhibition (interaction *p* = 0.406). When expressed as area under the curve, (Placebo: Week 0 vs. Week 12: 13,137 ± 2716 vs. 12,168 ± 2483; SGLT2 inhibition: Week 0 vs. Week 12: 13,122 ± 1984 vs. 11,879 ± 1966 mg/dL/min) glucose was lower during the week 12 OGTT (main effect *p* < 0.001), but there was no group difference (main effect *p* = 0.797) or influence of SGLT2 inhibition (interaction *p* = 0.653).

Fasting insulin was not different between groups (*p* = 0.251), was unchanged (*p* = 0.056) over 12 weeks, and SGLT2 inhibition did not influence the overall response (Placebo: 11.1 ± 3.5 vs. 6.5 ± 1.0; SGLT2 Inhibition: 6.9 ± 0.8 vs. 5.5 ± 0.7 mU/L/min; interaction *p* = 0.304). Circulating insulin concentration during the OGTT is presented in Figure 2B. On average, insulin was greater in the placebo group (main effect *p* = 0.032), and compared with week 0, insulin was lower during the week 12 OGTT (main effect *p* < 0.001); there was no influence of SGLT2 inhibition (interaction *p* = 0.382). When expressed as area under the curve, (Placebo: Week 0 vs. Week 12: 8966 ± 7321 vs. 7418 ± 6423; SGLT2 inhibition: Week 0 vs. Week 12: 8292 ± 5062 vs. 5693 ± 3376 mU/L/min) insulin was lower during the week 12 OGTT (main effect *p* < 0.001), but there was no group difference (main effect *p* = 0.435) or influence of SGLT2 inhibition (interaction *p* = 0.357). HOMA-IR was not different between groups (*p* = 0.30), was unchanged (*p* = 0.561) over 12 weeks, and SGLT2 inhibition did not influence the overall response (Placebo: 2.05 ± 3.00 vs. 1.20 ± 0.99; SGLT2 Inhibition: 1.30 ± 0.87 vs. 0.99 ± 0.52; interaction *p* = 0.551).

Fasting glucagon was not different between groups (*p* = 0.406), was unchanged (*p* = 0.13) over 12 weeks, and SGLT2 inhibition did not influence the overall response (interaction *p* = 0.388). Circulating glucagon concentration during the OGTT is presented in Figure 2C. On average, glucagon was greater in the SGLT2 inhibition group (main effect *p* = 0.023) but was unchanged over 12 weeks (main effect *p* < 0.582); SGLT2 inhibition did not influence this response (interaction *p* = 0.276). When expressed as area under the curve, (Placebo: Week 0 vs. Week 12: 465 ± 288 vs. 415 ± 246; SGLT2 inhibition: Week 0 vs. Week 12: 508 ± 298 vs. 535 ± 401 pmol/L/min) glucagon was similar between groups (main effect *p* = 0.344), unchanged over time (main effect *p* = 0.731) and the overall response was not influenced by SGLT2 inhibition (interaction *p* = 0.236).

The ratio of fasting insulin to fasting glucagon (i.e., insulin/glucagon) was not different between groups (*p* = 0.175), did not change over 12 weeks (*p* = 0.059), and SGLT2 inhibition did not influence the overall response (interaction *p* = 0.146). During the OGTT the ratio was higher in the placebo group (main effect *p* = 0.022) but was unchanged over 12 weeks (main effect *p* = 0.288); SGLT2 inhibition did not influence this response (interaction *p* = 0.369).

### 3.5. Lipid Profile

Circulating cholesterol and triglyceride concentrations are presented in Table 4. Dietary counselling decreased triglyceride, and very low-density lipoprotein cholesterol; the influence of SGLT2 inhibition on these responses did not attain statistical significance (*p* ≥ 0.05). Total cholesterol, high-density lipoprotein cholesterol, low-density lipoprotein cholesterol, and the ratio of total to high-density lipoprotein cholesterol were unchanged following dietary counselling. However, there were interactions with time and placebo vs. SGLT2 inhibition for high-density lipoprotein cholesterol, and the ratio of total to high-density lipoprotein cholesterol, driven by favorable modification with placebo and moderate unfavorable modification with SGLT2 inhibition.

### 3.6. Hunger and Appetite

Energy intake at the breakfast buffet and responses to the hunger and satiety questionnaire are presented in Table 5. Consistent with baseline dietary intake records (Table 1) participants assigned to the placebo condition appeared to eat more at the breakfast buffet (i.e., main effect of group). Following dietary counseling, fat ingestion at the breakfast buffet was decreased while carbohydrate, protein, and total caloric ingestion were unchanged. SGLT2 inhibition did not influence these responses.

Dietary counselling decreased sensations of hunger and satiety (questions 3 and 4) when assessed one hour after ingestion of the liquid primer meal. None of the other responses to the hunger and satiety questions at any given time were influenced by dietary counselling or SGLT2 inhibition (all *p* > 0.05).

## 4. Discussion

The purpose of the current study was to determine the efficacy of supplementing dietary counselling for weight loss with SGLT2 inhibition. We hypothesized that, compared with placebo, SGLT2 inhibition as an adjunct to dietary counselling would lead to greater magnitude of weight loss, more favorable modification of body composition, improved glucose regulation, and improved lipid profile. Our data indicate that 12 weeks of out-patient dietary counseling was associated with weight loss, fat loss, decreased resting metabolic rate, increased reliance on fat oxidation at rest, improved glucose regulation, and decreased circulating concentrations of triglyceride, and very low-density lipoprotein cholesterol; SGLT2 inhibition did not modify any of these responses. However, SGLT2 inhibition was associated with a greater decrease in fat-free mass, and a small decrease in plasma high-density lipoprotein cholesterol.

The average weight loss in our study was 3.9 kg over 12 weeks (~0.33 kg/week). This magnitude of weight loss is comparable to other studies of dietary counseling focusing on modest caloric restriction [35,36]. Similarly, this magnitude is also consistent with reports of weight loss mediated by SGLT2 inhibition. Based on recent reviews [15,16], SGLT2 inhibition evokes weight loss in the range of 1 to 5 kg, the precise magnitude of which appears to be partially dependent on the duration of the intervention, the dose and type of SGLT2 inhibitor, and the pre-intervention characteristics of the recipient(s) such as initial body mass and renal function [16]. In our study, there were no appreciable physiological differences between participants assigned to the placebo and SGLT2 inhibition treatments, including body mass, body composition, and estimated glomerular filtration rate. There were also no differences in baseline fasting blood glucose concentrations, and all of these concentrations, despite the sedentary and overweight/obese physical characteristics of the participants, were comfortably within the normoglycemic range (61–97 mg/dL). It is plausible that the glucosuria, and hence negative energy balance, mediated by SGLT2 inhibition was trivial in our diabetes-free study population. However, we do not believe this to be the case as several previous studies have demonstrated dapagliflozin mediated glucosuria (up to 80 mg/day) in healthy, diabetes-free adults [13,14]. Further, renal glucose reabsorption has a lower upper limit in diabetes-free adults [37], thus even in healthy men and women, SGLT2 inhibition lowers the threshold for urinary glucose excretion and evokes physiologically relevant glucosuria. In summary, our dietary counselling evoked weight loss that was in line with other dietary counselling studies, was comparable with other studies of SGLT2 mediated weight loss, but importantly, was unaffected by SGLT2 inhibition. A definitive physiological explanation for the absence of an additive effect of SGLT2 inhibition is currently elusive.

While diet-induced weight loss is often desirable, changes in body composition are usually of greater clinical relevance. In this regard, in our study, when SGLT2 inhibition was administered as an adjunct to dietary counselling, there was a greater decrease in fat-free mass (over one additional kg of lost lean tissue). Our observation is consistent with several previous studies that also report loss of fat-free mass with SGLT2 inhibition [15,38,39]. Noteworthy, in some of these studies, the duration of treatment was appreciably longer (up to 52 weeks) and the loss of fat-free mass did not become significant until week 24. It is feasible that, in our study, combining SGLT2 inhibition with dietary counselling may have accelerated the decrease in fat-free mass. In light of the importance of fat-free mass to metabolic health, appreciable loss is considered undesirable. Indeed, the unfavorable association between SGLT2 inhibition and sarcopenia (decreased skeletal muscle mass and/or quality/function) has been the subject of discussion within scientific literature [15,40]. It has been proposed that prolonged SGLT2 inhibition may promote sarcopenia via the combination of gluconeogenesis in the liver and increased proteolysis in skeletal muscle. In a previous study, we demonstrated decreased body mass and body fat, but maintenance of skeletal muscle mass when SGLT2 inhibition was combined with endurance exercise training [24]. One might suspect that the potential unfavorable effects of SGLT2 inhibition on skeletal muscle could also be attenuated with resistance exercise and/or targeted dietary modification (i.e., modest increases in protein intake).

In addition to weight loss and changes in body composition, we studied the influence of dietary counselling on glucose regulation. As expected, and consistent with other weight loss studies, glucose regulation (reflected by the lower areas under the glucose and insulin response curves during an OGTT) was improved. Unexpectedly, SGLT2 inhibition provided no additional benefit. One potential explanation for this observation pertains to glucagon secretion. SGLT2 inhibition has been suggested to promote endogenous glucose production in response to increased pancreatic secretion of glucagon [41,42], thus potentially masking, or blunting the benefits of SGLT2 inhibition. Our data, consistent with recent reports from animal studies of isolated perfused rat pancreas [43], and human studies describing inter-individual heterogeneity of SGLT2 expression and function in pancreatic islets [44], showed that glucagon concentration was unaffected by SGLT2 inhibition.

We hypothesized that some of the benefits associated with weight loss, such as the favorable modification of the lipid profile, would be further enhanced with SGLT2 inhibition. While some of the lipid profile parameters (e.g., triglyceride, and very low-density lipoprotein cholesterol) were favorably modified, contrary to our hypothesis, SGLT2 inhibition provided no additional benefit, and in some instances evoked a potentially unfavorable response such as a very modest decrease in high-density lipoprotein cholesterol. This observation is not without precedent; there have been previous reports of modest decreases in high-density lipoprotein cholesterol following 8-weeks of significant carbohydrate restriction (akin to SGLT2 inhibition) before increasing at 6 and 12 months [45]. The explanation for our observation is not obvious; we speculate that there may have been some contribution from the greater loss in fat-free mass with SGLT2 inhibition [46]. Lipoprotein lipase is synthesized by skeletal muscle and bound to skeletal muscle capillaries. It is feasible that the greater muscle loss led to lower bioavailable lipoprotein lipase and therefore lower generation of high-density lipoprotein cholesterol [47,48]. Direct assessment of lipoprotein lipase activity would be necessary to confirm or refute this explanation. This explanation also provides indirect support for combining exercise with dietary counseling for weight loss when co-administered with SGLT2 inhibition.

An additional aim of our study was to explore the effects of SGLT2 inhibition and dietary counselling on dietary preferences and hunger and appetite prior to and following acute feeding. Our rationale was based on the influence of SGLT2 inhibition on the absorption of dietary carbohydrates. In light of the increased glucosuria, the SGLT2 inhibited state is unique in that dietary carbohydrate restriction is not required; patients receiving SGLT2 inhibitors may ingest but are unable to fully utilize carbohydrates. Essentially, the SGLT2 inhibited state is akin to a modest carbohydrate-restricted mimetic. Noteworthy, the SGLT2 inhibited state has also been discussed in the context of a ketogenic diet [49,50] and our current and previous data demonstrate greater reliance on fat oxidation at rest and during exercise [24]. We surmised that low carbohydrate absorption may be “sensed” and eventually lead to adaptation manifested as changes in eating behavior. In a recent study, the influence of short-term (2-weeks) SGLT2 inhibition on dietary preferences (i.e., increased desire for sweet foods) and appetite were quantified; no *statistically* significant effects were observed [23]. In the current study, we extended the period of SGLT2 inhibition to 12 weeks and hypothesized that this longer duration of treatment would evoke measurable effects on dietary preferences, and hunger and appetite. Contrary to our hypothesis, and in agreement with the previous study [23], we observed no influence of SGLT2 inhibition on scores of hunger and appetite, and on eating behavior (i.e., no differences in quantities and types of food selected and ingested). Hyperphagia has been proposed as one of the mechanisms to explain lower than predicted weight loss (based on caloric loss mediated by glucosuria) reported during SGLT2 inhibition [51]. Our data, together with recent observations, refute this assertion. Further, data provided from our “surprise” requests for dietary recall during weeks 5 and 10 of our intervention provide additional evidence that SGLT2 inhibition, when combined with dietary counselling, did not change eating behavior in a manner different to those assigned to the placebo adjunct.

Our study and design have several potential limitations that warrant additional consideration. The purpose of the study was to determine the efficacy of supplementing dietary counselling for weight loss with SGLT2 inhibition, and the hypothesis was developed from the perspective of how SGLT2 inhibition might influence physiological adaptation to weight loss. However, it is possible that our question was misplaced, and instead we should have asked how weight loss influences SGLT2 inhibition. It is feasible that the responses to a pharmaceutical prescribed to decrease macronutrient (carbohydrate) absorption may become redundant when co-administered with a prescription for caloric restriction. To fully address this question, a four-square approach would have been necessary: placebo with/without dietary counselling vs. SGLT2 inhibition with/without dietary counselling. Unfortunately, this design was beyond the scope of our resources.

The total weight loss mediated by dietary counselling was not influenced by SGLT2 inhibition. In addition to decreased dietary intake (or absorption), weight loss is also mediated by increased physical activity and habitual exercise. Although the decrease in resting energy expenditure was similar between the placebo and SGLT2 inhibition treatments, it may have been useful to have assessed the other components of total daily energy expenditure: the thermic effects of feeding and physical activity. At this time, we are unaware of any evidence to indicate SGLT2 inhibition has appreciable effects on either of these other components. Further, although two previous studies have already described dapagliflozin-mediated glucosuria (up to 80 mg/day) in healthy, diabetes-free adults [13,14], the addition of these measurements in the current study would have provided useful, additional insight.

Finally, our total sample size (*n* = 50) might be considered small, thus it is plausible that with greater statistical degrees of freedom, we may have identified additional interactions between SGLT2 inhibition and dietary counselling. However, with the exception of two of the lipid profile parameters (triglyceride, and very low-density lipoprotein cholesterol; Table 4) none of the other reported interactions came close to meeting the conventional level of statistical significance (*p* < 0.05). Further, even with a relatively small sample size, we were able to identify statistical interactions between SGLT2 inhibition and dietary counselling pertaining to fat-free mass and high-density lipoprotein cholesterol. Thus, we suggest that from the dependent variables quantified in the current study, with the exception of two lipid profile parameters, only two dependent variables were subject to SGLT2 inhibition and dietary counselling interaction.

## 5. Conclusions

Twelve weeks of out-patient dietary counseling was associated with weight loss, fat loss, decreased resting metabolic rate, increased reliance on fat oxidation at rest, improved glucose regulation, and decreased circulating concentrations of triglyceride, and very low-density lipoprotein cholesterol; SGLT2 inhibition did not modify any of these responses. However, SGLT2 inhibition was associated with a greater decrease in fat-free mass, and a small decrease in plasma high-density lipoprotein cholesterol. In light of the health implications of the modest decreases in fat-free mass and circulating high-density lipoprotein cholesterol, we recommend careful consideration before implementing SGLT2 inhibition as an adjunct to dietary counselling for weight loss in sedentary adults with obesity.

## Figures and Tables

**Table 1:** Selected baseline physiological and dietary characteristics.

Variable	Placebo	SGLT2 Inhibition	*p*-Value
Male/Female	7/18	5/20	-
Age (years)	35 ± 11	38 ± 11	0.362
Height (cm)	167 ± 9	169 ± 8	0.399
Body Mass (kg)	93.8 ± 20.4	96.5 ± 15.6	0.388
Body Mass Index (kg/m^2^)	33.2 ± 5.6	33.5 ± 3.9	0.858
Blood Pressure (mmHg)	112/69 ± 8/5	116/69 ± 10/8	0.066/0.819
VO_2peak_ (mL/kg/min)	24.8 ± 5.6	22.2 ± 5.0	0.100
Fasting Glucose (mg/dL)	74.7 ± 6.9	76.1 ± 7.7	0.508
Fasting Insulin (mU/L)	5.1 ± 2.5	7.0 ± 3.9	0.120
Dietary Intake (kcal/day)	2187 ± 541	1819 ± 491	0.015
Carbohydrate (g/day)	246 ± 73	197 ± 59	0.012
Fat (g/day)	91 ± 28	77 ± 26	0.098
Protein (g/day)	85 ± 24	80 ± 25	0.190
eGFR (mL/min/1.73 m^2^)	152 ± 54	154 ± 52	0.881

Data are mean ± SD. VO_2peak_: Peak oxygen uptake. eGFR: Estimated glomerular filtration rate. Statistical *P*-values are derived from one-way analysis of variance.

**Table 2:** Dietary intake following 5, and 10 weeks of dietary counselling for weight loss supplemented with placebo or SGLT2 inhibition.

Variable	Placebo		SGLT2 Inhibition		ANOVA *p*-Values		
	Week 5	Week 10	Week 5	Week 10	Group	Time	G × T
Total(kcal)	1586 ± 386	1644 ± 484	1691 ± 672	1544 ± 444	0.983	0.633	0.269
Pro (g)	74 ± 29	82 ± 40	74 ± 31	78 ± 36	0.828	0.323	0.761
CHO (g)	167 ± 61	169 ± 78	186 ± 79	160 ± 62	0.721	0.337	0.268
Fat (g)	68 ± 23	70 ± 24	72 ± 39	65 ± 31	0.990	0.660	0.388
Pro (%)	19 ± 8	20 ± 8	18 ± 6	20 ± 7	0.768	0.174	0.526
CHO (%)	42 ± 10	41 ± 13	44 ± 9	41 ± 11	0.488	0.286	0.619
Fat (%)	38 ± 10	39 ± 11	37 ± 9	38 ± 11	0.609	0.796	0.881

Data are mean ± SD and reflect average daily intake based on recall. Total: total dietary intake. Pro: Protein. CHO: Carbohydrate. ANOVA: Analysis of variance. G × T: Group-by-Time interaction.

**Table 3:** Body mass and composition prior to and following 12 weeks of dietary counselling for weight loss supplemented with placebo or SGLT2 inhibition.

Variable	Placebo		SGLT2 Inhibition		ANOVA *p*-Values		
	Baseline	Week 12	Baseline	Week 12	Group	Time	G × T
Mass (kg)	93.8 ± 20.4	90.3 ± 19.7	96.5 ± 15.6	92.1 ± 16.7	0.660	<0.001	0.394
BMI (kg/m^2^)	33.2 ± 5.6	32.0 ± 5.4	33.5 ± 3.9	32.0 ± 4.3	0.936	<0.001	0.446
WC (cm)	92.3 ± 10.2	90.7 ± 8.2	95.6 ± 9.0	94.2 ± 10.8	0.239	0.052	0.902
Fat Mass (kg)	37.0 ± 11.4	35.0 ± 11.2	40.1 ± 9.0	37.9 ± 10.3	0.306	<0.001	0.770
Body Fat (%)	39.8 ± 6.0	38.7 ± 6.8	41.8 ± 5.2	41.1 ± 6.0	0.202	0.001	0.385
FFM (kg)	55.3 ± 11.2	54.6 ± 11.4	55.5 ± 9.2	53.6 ± 9.3	0.897	<0.001	0.047
BMC (kg)	2.4 ± 0.4	2.5 ± 0.4	2.6 ± 0.3	2.6 ± 0.3	0.181	0.027	0.807

Data are mean ± SD. BMI: Body mass index. WC: Waist circumference. FFM: Fat-free mass. BMC: Bone mineral content. ANOVA: Analysis of variance. G × T: Group-by-Time interaction.

**Table 4:** Lipid profile prior to and following 12 weeks of dietary counselling for weight loss supplemented with placebo or SGLT2 inhibition.

Variable	Placebo		SGLT2 Inhibition		ANOVA *p*-Values		
	Baseline	Week 12	Baseline	Week 12	Group	Time	G × T
TC	177 ± 30	173 ± 27	170 ± 28	162 ± 24	0.244	0.060	0.593
HDLC	46 ± 11	48 ± 11	48 ± 13	46 ± 14	0.982	0.924	0.028
TG	148 ± 65	121 ± 53	127 ± 55	125 ± 50	0.557	0.027	0.055
nHDLC	131 ± 31	125 ± 27	122 ± 29	116 ± 24	0.250	0.036	0.929
TC:HDLC	4.0 ± 1.1	3.8 ± 0.9	3.7 ± 1.0	3.8 ± 1.0	0.641	0.052	0.021
LDLC	101 ± 26	101 ± 22	97 ± 24	91 ± 19	0.260	0.184	0.320
VLDLC	30 ± 13	24 ± 11	25 ± 11	25 ± 10	0.561	0.026	0.050

Data are mean ± SD. All units are mg/dL. TC: Total Cholesterol. HDLC: High-density lipoprotein cholesterol. TG: Triglyceride. nHDLC: HDLC subtracted from TC. LDLC: Low-density lipoprotein cholesterol. VLDLC: Very low-density lipoprotein cholesterol. ANOVA: Analysis of variance. G × T: Group-by-Time interaction.

**Table 5:** Energy intake at breakfast buffet and responses to satiety questionnaire prior to and following 12 weeks of dietary counselling for weight loss supplemented with placebo or SGLT2 inhibition.

	Placebo		SGLT2 Inhibition		ANOVA *p*-Values		
	Week 0	Week 12	Week 0	Week 12	Group	Time	G × T
Energy Intake							
Fat	163 ± 70	132 ± 64	122 ± 99	92 ± 64	0.028	0.011	0.977
CHO	426 ± 187	431 ± 235	345 ± 130	297 ± 141	0.018	0.389	0.290
Protein	119 ± 38	114 ± 62	96 ± 51	82 ± 33	0.022	0.169	0.512
Total	693 ± 208	660 ± 313	549 ± 209	458 ± 184	0.004	0.059	0.381
Responses to Satiety Questionnaire							
Baseline							
Q1	49 ± 26	52 ± 29	37 ± 30	37 ± 30	0.069	0.735	0.646
Q2	49 ± 25	50 ± 29	35 ± 29	35 ± 28	0.041	0.892	0.892
Q3	20 ± 14	23 ± 17	26 ± 23	22 ± 18	0.583	0.994	0.215
Q4	50 ± 14	53 ± 23	49 ± 25	42 ± 22	0.247	0.571	0.098
Q5	51 ± 19	57 ± 22	56 ± 24	60 ± 22	0.414	0.213	0.658
Post-Liquid Meal Primer (0 min)							
Q1	47 ± 24	46 ± 26	34 ± 24	32 ± 26	0.023	0.816	0.789
Q2	47 ± 24	48 ± 25	35 ± 26	29 ± 24	0.010	0.678	0.240
Q3	25 ± 14	30 ± 15	30 ± 21	35 ± 21	0.216	0.089	0.951
Q4	49 ± 18	51 ± 22	47 ± 22	39 ± 25	0.160	0.570	0.054
Q5	45 ± 17	52 ± 22	48 ± 24	54 ± 23	0.687	0.082	0.884
Post-Liquid Meal Primer (60 min)							
Q1	54 ± 19	48 ± 24	44 ± 24	38 ± 21	0.077	0.075	0.900
Q2	53 ± 18	53 ± 21	42 ± 25	39 ± 20	0.016	0.565	0.663
Q3	21 ± 12	28 ± 16	27 ± 16	31 ± 18	0.237	0.044	0.526
Q4	57 ± 13	51 ± 20	52 ± 17	44 ± 19	0.152	0.015	0.582
Q5	48 ± 18	53 ± 21	59 ± 20	58 ± 19	0.103	0.446	0.276
Post-Breakfast Buffet (0 min)							
Q1	14 ± 14	11 ± 10	8 ± 9	12 ± 8	0.388	0.768	0.193
Q2	12 ± 12	11 ± 11	6 ± 6	8 ± 7	0.033	0.870	0.468
Q3	68 ± 15	67 ± 20	72 ± 14	72 ± 18	0.271	0.871	0.782
Q4	17 ± 14	13 ± 10	14 ± 15	14 ± 14	0.762	0.333	0.551
Q5	34 ± 21	37 ± 23	41 ± 24	42 ± 22	0.271	0.561	0.831
Post-Breakfast Buffet (60 min)							
Q1	15 ± 15	15 ± 14	11 ± 8	11 ± 9	0.158	0.795	0.952
Q2	12 ± 9	14 ± 15	10 ± 9	10 ± 9	0.290	0.563	0.689
Q3	62 ± 19	63 ± 19	57 ± 22	56 ± 21	0.218	0.929	0.780
Q4	20 ± 14	19 ± 17	21 ± 17	19 ± 16	0.854	0.373	0.845
Q5	38 ± 19	43 ± 22	40 ± 18	48 ± 17	0.409	0.03	0.585
Post-Breakfast Buffet (120 min)							
Q1	22 ± 16	24 ± 14	22 ± 18	15 ± 13	0.265	0.345	0.044
Q2	20 ± 15	20 ± 14	21 ± 14	15 ± 10	0.425	0.222	0.235
Q3	54 ± 20	56 ± 17	52 ± 22	49 ± 20	0.336	0.938	0.429
Q4	20 ± 19	24 ± 14	30 ± 18	28 ± 18	0.624	0.211	0.603
Q5	43 ± 21	44 ± 20	46 ± 18	50 ± 18	0.528	0.372	0.74
Post-Breakfast Buffet (180 min)							
Q1	35 ± 20	36 ± 19	33 ± 24	25 ± 20	0.200	0.249	0.116
Q2	35 ± 20	36 ± 20	34 ± 25	25 ± 21	0.254	0.120	0.105
Q3	43 ± 15	48 ± 18	41 ± 20	40 ± 20	0.205	0.499	0.324
Q4	40 ± 13	36 ± 18	40 ± 19	33 ± 21	0.645	0.053	0.693
Q5	47 ± 21	47 ± 23	52 ± 17	56 ± 18	0.149	0.511	0.561

Data are mean ± SD. Units for energy intake: kcal. Units for responses to satiety questions: mm. At baseline, 0 and 60 min after liquid meal primer (i.e., pre-load), and 0, 60, 120, and 180 min post-breakfast buffet, participants responded to five questions pertinent to hunger and satiety: (**Q1**) How strong is your desire to eat right now? (**Q2**) How hungry do you feel right now? (**Q3**) How full do you feel right now? (**Q4**) How much do you think you could eat right now? (**Q5**) How thirsty do you feel right now? Participants responded to these questions via a 100 mm visual analog scale, where 0 was “negligible” and 100 was “maximal”. ANOVA: Analysis of variance. G × T: Group-by-Time interaction. Main effects of time relative to meal ingestion are not displayed.
